# Clinical and radiological outcomes of jumbo cup in revision total hip arthroplasty: A systematic review

**DOI:** 10.3389/fsurg.2022.929103

**Published:** 2022-10-04

**Authors:** Qiuyuan Wang, Qi Wang, Pei Liu, Juncheng Ge, Qidong Zhang, Wanshou Guo, Weiguo Wang

**Affiliations:** ^1^ Graduate School, Beijing University of Chinese Medicine, Beijing, China; ^2^ Department of Orthopaedic Surgery, China-Japan Friendship Hospital, Beijing, China; ^3^ Department of Orthopaedic Surgery, Peking University of China-Japan Friendship School of Clinical Medicine, Beijing, China; ^4^ Department of Adult Joint Reconstruction, Henan Luoyang Orthopedic Hospital (Henan Provincial Orthopedic Hospital), Zhengzhou, China

**Keywords:** jumbo cup, acetabular bone defect, revision total hip arthroplasty, survivorship, rotation hip center

## Abstract

**Introduction:**

Many studies have reported the clinical outcomes of a jumbo cup in revision total hip arthroplasty (rTHA) with acetabular bone defect. We conducted a systematic review to access the survivorship and clinical and radiological outcomes of a jumbo cup in rTHA.

**Methods:**

A systematic review was conducted according to the Preferred Reporting Items for Systematic Reviews and Meta-Analyses guidelines. A comprehensive literature search from PubMed, MEDLINE, EMBASE, and the Cochrane Database of Systematic Reviews was performed with the keywords (“revision” OR “revision surgery” OR “revision arthroplasty”) AND (“total hip arthroplasty” OR “total hip replacement” OR “THA” OR “THR”) AND (“jumbo cup” OR “jumbo component” OR “extra-large cup” OR “extra-large component”). Studies reporting the clinical or radiological outcomes were included. The basic information and radiological and clinical results of these studies were extracted and summarized for analysis.

**Results:**

A total of 19 articles were included in the systematic review. The analysis of clinical results included 953 hips in 14 studies. The re-revision-free survivorship of the jumbo cup was 95.0% at a mean follow-up of 9.3 years. Dislocation, aseptic loosening, and periprosthetic joint infection were the top three complications with an incidence of 5.9%, 3.0%, and 2.1%, respectively. The postrevision hip center was relatively elevated 10.3 mm on average; the mean postoperative leg-length discrepancy was 5.4 mm.

**Conclusion:**

A jumbo cup is a favorable option for acetabular bone defect reconstruction in rTHA with satisfying survivorship and acceptable complication rates.

## Introduction

Reconstruction of an acetabular bone defect is a difficult procedure in revision total hip arthroplasty (rTHA) (1). In rTHA, the primary goal of acetabular reconstruction is creating sufficient mechanical support and bone contact for the acetabular cup, thereby achieving bone ingrowth or ongrowth and attaining stability of the acetabular cup (2). In addition, the position of the rotation hip center is also an important factor to consider. Due to the complexity and variety of the acetabular bone defect, it is highly possible for unexpected challenges to occur beyond the preoperative plan. Moreover, many patients who accept revision arthroplasty are at an advanced age with underlying diseases; therefore, there is need for enhancing surgical efficiency and limiting damage control. These factors all bring significant challenges to surgeons.

Before performing rTHA, an evaluation of the acetabular bone defect is necessary. The Paprosky classification, American Academy of Orthopaedic Surgeons (AAOS) classification, and Gross system are the most commonly used in preoperative planning (3, 4). Many new methods of evaluation have also been developed to help surgeons make better surgical strategy (5–9). Currently, the Paprosky type IIIA and IIIB and AAOS type III and IV are regarded as the most challenging conditions, which involve extensive bone loss of the acetabular rim and columns, and even pelvic discontinuity (10, 11). Several methods of reconstruction have been developed for severe acetabular bone loss in rTHA, such as structural allografts, impaction bone grafting, the jumbo cup, the highly porous metallic augments and hemispherical cup, the cup-cage system, custom monoflanged acetabular components, and the cup-on-cup technique (12–20). Many studies as well as systematic reviews have reported the clinical results of these reconstruction techniques (21–24), but the most effective solution remains controversial.

Using a jumbo cup is one of the most commonly recommended reconstruction methods for severe acetabular bone defect in rTHA. It was first reported by Jasty in 1998 (25). There is no strict definition for the jumbo cup. Most papers define the jumbo cup as a diameter of over 66 or 64 mm for males and over 62 or 60 mm for females. The advantages of the jumbo cup include the obvious simplified surgical procedure, more contact area with the host bone, and less requirement for bone graft (26). However, there are also some limitations in using the jumbo cup for reconstruction. For example, it may result in further bone loss (27), which may delay the full weight-bearing time and even cause a protrusion of the jumbo cup into the pelvic cavity. Therefore, the jumbo cup is usually applied in Paprosky type I–III acetabular bone defect and rarely used alone for pelvic discontinuity (11). In many cases, jumbo cups have to be set in a high position to provide sufficient contact with the host bone and mechanical support, which may lead to rotation center elevation, leg-length discrepancy (LLD), and soft tissue imbalance (28). Although many studies have reported the clinical results of the jumbo cup in rTHA, no systematic review has been conducted to date. In this context, the aim of this study is to systematically summarize the current evidence of the jumbo cup in rTHA, including survivorship and failure, radiological outcome, hip function, and complications.

## Materials and methods

### Literature search strategy

We conducted a comprehensive literature search from the electronic databases PubMed, EMBASE, Web of Science, and Cochrane Library. The last literature search was on 15 April 2022. The search project was based on the following keywords: (“revision” OR “revision surgery” OR “revision arthroplasty”) AND (“total hip arthroplasty” OR “total hip replacement” OR “THA” OR “THR”) AND (“jumbo cup” OR “jumbo component” OR “extra-large cup” OR “extra-large component”). The language was limited to English. If the abstract was not sufficient for us to include or exclude a study, we would download the full text. The literature search process was carried out on the basis of the Preferred Reporting Items for Systematic Reviews and Meta-Analyses (PRISMA) guidelines (29).

### Inclusion and exclusion criteria

Only those clinical studies that met the following criteria were included in this systematic review: (1) rTHA with acetabular bone defect; (2) using a jumbo cup for acetabular reconstruction; and (3) reporting clinical results or radiological data. Reviews, conference abstracts, non-English written articles, letters, case reports, experiment studies, and simulation studies were excluded from this systematic review.

### Data extraction

Two researchers separately extracted all data according to the rules described above. We developed an extraction table for data extraction including the following: (1) basic information of each study; (2) radiological results evaluating the implant position; and (3) clinical results including studying the accuracy of the preoperative plan, intraoperative details, and postoperative function complications. All data were extracted by two investigators; any disagreement was solved by an expert surgeon and a third researcher to make a final decision.

### Quality assessment

We used the Methodological Index for Non-Randomized Studies (MINORS) for quality assessment (30). This evaluation system involves 12 items for comparative studies and 8 items for noncomparative studies, with total scores of 24 and 16, respectively. The item was separately scored with 0, 1, and 2 corresponding to nonreported information, inadequate information, and adequate information, respectively. Two authors independently filled the evaluation system. Studies that scored >75% of the total score were considered to have a low risk of bias.

## Results

### Literature selection

**Figure 1:**
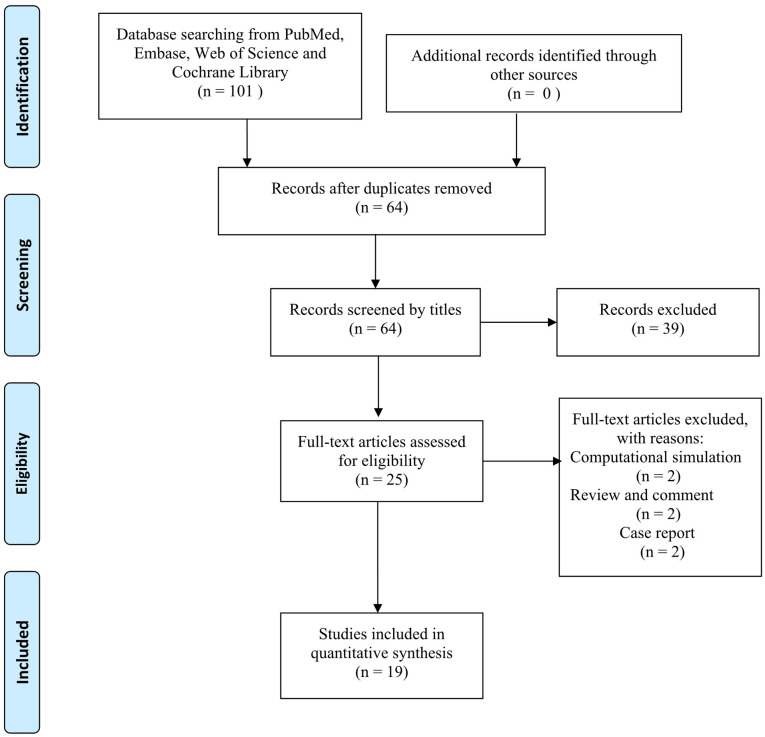
PRISMA flow diagram.

The literature search initially identified 101 articles and finally included 19 articles in this systematic review according to the inclusion and exclusion criteria (31–49) (Figure 1). Among the 19 articles, 2 articles were pure radiological studies (31, 40). There were three pairs of cognate articles (six articles) that were published in different years (38, 39, 41, 45, 48, 49). For these coupled cognate articles, the results of the more recent articles were adopted.

### Quality assessment

Except for the two pure radiological studies, the remaining 17 clinical studies were included in the quality assessment (32–39, 41–49) (Table 1). Only one study was a comparative study and its quality was high (21/24) (33). The other 16 studies were noncomparative studies, with a mean MINORS score of 10.5, which indicated that the general quality of these studies was relatively low. Therefore, a further meta-analysis was not conducted.

**Table 1:** MINORS scale.

Author/year	Clearly stated study aim	Inclusion of consecutive patients	Prospective collection of data	Appropriate endpoints	Unbiased assessment of endpoint	Appropriate follow-up period	Loss to follow-up less than 5%	Prospective calculation of study size	Adequate control group	Contemporary groups	Baseline equivalence of groups	Adequate statistical analyses	Total score
Zhang 2019	2	2	2	2	1	2	0	0	NA	NA	NA	NA	9/16
Zhou 2018	2	2	2	2	1	2	2	0	2	2	2	2	21/24
Salem 2018	2	1	2	2	0	2	2	0	NA	NA	NA	NA	11/16
Moon 2018	2	1	2	2	0	2	2	0	NA	NA	NA	NA	11/16
McLaughlin 2018	2	2	2	2	0	2	0	0	NA	NA	NA	NA	10/16
Jo 2016	2	1	2	2	0	2	2	0	NA	NA	NA	NA	11/16
von Roth 2015	2	2	2	2	0	2	0	0	NA	NA	NA	NA	10/16
Gustke 2014	2	2	2	2	0	2	2	0	NA	NA	NA	NA	12/16
Lachiewicz 2013	2	2	2	2	1	2	1	0	NA	NA	NA	NA	12/16
Wedemeyer 2008	2	1	2	2	0	2	2	0	NA	NA	NA	NA	11/16
Fan 2008	2	2	2	2	1	1	2	0	NA	NA	NA	NA	12/16
Hendricks 2006	2	1	2	2	0	2	0	0	NA	NA	NA	NA	9/16
Gustke 2004	2	1	2	2	0	2	0	0	NA	NA	NA	NA	9/16
Patel 2003	2	1	2	1	0	2	2	0	NA	NA	NA	NA	10/16
Obenaus 2003	2	1	2	2	0	2	0	0	NA	NA	NA	NA	9/16
Whaley 2001	2	2	2	2	0	2	2	0	NA	NA	NA	NA	12/16
Dearborn 2000	2	2	2	2	0	2	0	0	NA	NA	NA	NA	10/16

MINORS, Methodological Index for Non-Randomized Studies.

### Demographics and characteristics

A total of 1,406 hips were initially included in the review (Table 2). To analyze the clinical results, the two pure radiological studies were removed and the results of the cognate articles were merged and adjusted. As a result, 953 hips in 14 studies were included in the analysis of clinical results (32–39, 41–44, 46, 47) (Tables 3–5). The patients who underwent rTHA had a mean age of 62.5 years. The mean follow-up was 9.3 years. In the radiological result analysis part, 631 hips in 11 studies were included (31–34, 37, 40, 43, 44, 47–49). The data of the hip center position and leg-length discrepancy were extracted and analyzed (Table 6).

**Table 2:** Characteristics and patient demographics of included studies.

Author/Year	Definition of jumbo cup	Study design	Hips of initial/final cohort	Patients	Gender (M/F)	Age (y)	BMI	Follow-up	Acetabular bone defect classification	Revision reason
Peng 2021	Male: ≥64 mm; Female: ≥60 mm	Pure radiological	88/88	88	44/44	61 ± 11 (32–85)	NA	Postoperative 1 week	NA	NA
Zhang 2019	Male: ≥64 mm; Female: ≥60 mm	Retrospective	73/63	61	29/32	59.4 ± 11.4	24.9 ± 3.8	5.7 years (2–16)	Paprosky type IIA 16, IIB 9, IIC 24, IIIA 8, IIIB 6	Aseptic loosening 55, second stage of a two-stage revision for PJI 6, wear and osteolysis 2
Zhou 2018	Male: ≥64 mm; Female: ≥60 mm	Retrospective comparative study	80/77	77	43/34	60.6 ± 10.4	23.7 ± 3.4	52 months (24–104)	Paprosky type IIA 18, IIB 12, IIC 25, IIIA 14, IIIB 8	Aseptic loosening 61, PJI 6, fracture 1, dislocation 2, others 7
Salem 2018	>60 mm	Case series	17/17	17	9/8	52 (40–61)	NA	3.5 years (2–6)	Paprosky type IIB 4, IIIA 13	Aseptic loosening all
Moon 2018	Male: ≥66 mm; Female: ≥62 mm	Retrospective	85/80	80	47/33	57.7 (30–78)	24.3 (19.2–28.3)	10.4 years (5–16.1)	Paprosky type IIA 16, IIB 17, IIC 22, IIIA 19, IIIB 6	Aseptic all
McLaughlin 2018	Male: ≥66 mm; Female: ≥62 mm	Case series	61/30	28	14/14	71 (36–79)	33	13 years (10–16)	Paprosky type I 2, IIA 4, IIB 11, IIC 12, IIIA 1	Aseptic loosening 26, recurrent dislocation 3, severe osteolysis 1
Jo 2016	>10 mm than templated contralateral hip	Retrospective	60/51	51	22/29	60.7 (30–81)	23.2 (15.3–36.1)	51 months (12–154)	Paprosky type IIA 8, IIB 19, IIC 13, IIIA 11	Aseptic loosening 39, second stage of a two-stage revision for PJI 5, acetabular protrusion 5, recurrent dislocation 2
von Roth 2015	Male: ≥66 mm; Female: ≥62 mm	Retrospective	89/89	89	46/43	59 (30–83)	30 (19–37)	Clinical: 20 years (14–27); Radiographic; 19 years (10–25)	Paprosky type I 6, IIA 11, IIB 26, IIC 17, IIIA 25, IIIB 4	Acetabular loosening 38, aseptic acetabular and femoral loosening 42, aseptic acetabular loosening with femoral periprosthetic fracture 5, other indications 4
Nwankwo 2014	Male: ≥66 mm; Female: ≥62 mm	Pure radiological	98/98	98	57/41	62.4 ± 12.2	NA	Radiographic: 6 weeks	NA	NA
Gustke 2014	Male: ≥66 mm; Female: ≥62 mm	Retrospective	216/199	189	71/118	66	NA	10 years (2–19)	Paprosky type I 14, IIA 52, IIB 65, IIC 18, IIIA 34, IIIB 16	Aseptic loosening of the acetabular component 148, septic loosening 21, recurrent dislocations 18, failed bipolar arthroplasties 4, persistent pain 2, excessive polyethylene wear patterns 3
									AAOS type I 8, II 74, III 115; pelvic discontinuity 2	
Lachiewicz 2013	Male: ≥66 mm; Female: ≥62 mm	Retrospective	129/108	101	52/49	63 (33–88)	27 (16–41)	8.1 (2–22)	Paprosky type I 1, IIA 22, IIB 23, IIC 5, IIIA 40, IIIB 17	Painful aseptic loosening 89, PJI 10, periprosthetic femoral fracture 4, polyethylene wear 3, recurrent dislocation 1, chronic dislocation with periprosthetic femoral fracture 1
Wedemeyer 2008	≥64 mm	Retrospective	17/17	17	10/7	60 (44–78)	NA	82 months (33–149)	Paprosky type IIA 5, IIB 3, IIC 4, IIIA 5	Aseptic prosthesis loosening all
									AAOS type II 12, III 5	Primary hip revision surgery 9, second revision 7, third revision 1
Fan 2008	Male: ≥64 mm; Female: ≥60 mm	Retrospective	50/47	46	23/23	61.4 (23–79)	NA	65 months (48–84)	Paprosky type I 6, IIA 13, IIB 11, IIC 6, IIIA 5, IIIB 6	Loosening of the acetabular cups (septic 2 and aseptic 42), insert wear 3
Hendricks 2006	>65 mm	Retrospective	24/12	12	NA	NA	NA	13.9 years (12.3–16.2)	NA	NA
Gustke 2004	Male: ≥66 mm; Female: ≥62 mm	Case series	166/166	NA	NA	NA	NA	6.1 years	Paprosky type I 10, II 119, III 37;	NA
									AAOS type II combined cavitary and segmental defects >105	
Patel 2003	Male: ≥66 mm; Female: ≥62 mm	Retrospective	43/43	42	NA	63 (25–86)	29 (21–42)	10 years (6–14)	Paprosky type IIA 21, IIB 6, IIC 10, IIIA 6	Aseptic loosening 29, wear and osteolysis 7, part of a two-stage revision PJI 2, failed resurfacing procedures 5
									AAOS type I 9, II 11, III 23	
Obenaus 2003	≥60 mm	Retrospective	99/60	59	NA	65.2 (39.8–79.5)	NA	5.6 years (4.1–7.1)	Paprosky type I 2, IIA 12, IIB 28, IIC 7, IIIA 9, IIIB 2	Aseptic cup loosening with enlargement of the acetabulum
Whaley 2001	Male: ≥66 mm; Female: ≥62 mm	Retrospective	89/89	89	46/43	59 (30–83)	30 (19–37)	7.2 years (5–11.3)	Paprosky type I 6, IIA 11, IIB 26, IIC 17, IIIA 25, IIIB 4;	Acetabular loosening 38, aseptic acetabular and femoral loosening 42, aseptic acetabular loosening with femoral periprosthetic fracture 5, other indications 4
									AAOS type I 3, II 10, III 76,	
Dearborn 2000	≥66 mm	Case series	24/24	24	18/6	58 (21–81)	NA	7 years (5–10.3)	AAOS type I 7, II 5, III 12	NA

BMI, body mass index; AAOS, American Academy of Orthopaedic Surgeons; PJI, periprosthetic joint infection.

**Table 3:** Additional procedures, survival, and Harris Hip Score.

Study	No. of hips	Additional procedure	Survival	Harris Hip Score	
				Preoperative	Postoperative
Zhang 2019	63	Morselized allografting for acetabular protrusion	16 years: 96.8% (EP = reoperation)	46	83
Zhou 2018	77	Bulk allograft (2 hips) and impaction bone grafting (12 hips) was used in Paprosky type III acetabular bone loss	4 years: 94.2% (EP = radiological or clinical failure)	46.7 ± 13.2	83.1 ± 9.0
Salem 2018	17	Particulate bone grafting from the iliac crest	No re-revision at last follow-up (mean 3.5 years)	42 (24–59)	85 (72–92)
Moon 2018	80	Structural bone allograft (4 hips), morselized bone allograft (47 hips)	16 years: 85% (worst); 91% (best)	53	77
McLaughlin 2018	30	Packing acetabular deficiencies with allograft bone chips and local bone obtained from reaming	16 years: 92.6% (EP = re-revision); 97.4% (EP = cup aseptic loosening)	49 (37–59)	86 (64–94)
Jo 2016	51	Autogenic (ipsilateral iliac crest) or allogenic (fresh-frozen chip bone) morselized bone graft for medial cavitary bone defect	13 years: 86.3% (EP = implant failure)	NA	No rim fixation: 75 ± 7.6 Rim fixation: 85 ± 8.5
von Roth 2015 and Whaley 2001	89	Particulate bone grafting (54 hips), and bulk bone grafting (9 hips)	20 years: 83% (free from any acetabular revision), 88% (free from aseptic loosening of the metal acetabular component), and 85% (free from aseptic or radiographic definite loosening of the metal acetabular component)	56	71 (30–95)
Gustke 2014 and Gustke 2004	216	Particulate autografting (51 hips), particulate allografting (49 hips), bulk allografting (38 hips)	4 years: 98%; 16y:96% (acetabular component)	44	72
Lachiewicz 2013	108	Bone grafting (108 hips): crushed cancellous allograft only (98 hips), iliac crest autograft (4 hips), both allograft and autograft (5 hips)	10 years: 97.3%, 15 years: 82.8% (EP = either acetabular cup revision for aseptic loosening or definite radiographic evidence of loosening); 10 years: 93.8%, 15 years: 79.8% (EP = acetabular cup removal for any reason); 10 years: 88.5%, 15 years: 56.5% (EP = any reoperation involving the hip)	NA	
Wedemeyer 2008	17	Morselized bone graft (15 hips)	NA	62	83
Fan 2008	47	Allograft bone graft (25 patients)	5 years: 94.5% (EP = implant failure)	NA	
Hendricks 2006 and Dearborn 2000	24	Particulate autologous or allograft bone packing acetabular defects	NA	54 (31–82)	79 (46–98)
Patel 2003	43	Bulk allograft (8 hips), morselized allograft alone (27 hips)	Acetabular shell: 14 years: 92%; acetabular shell: 13 years: 83%	48 ± 15	81 ± 18
Obenaus 2003	60	Press-fit structural allografting (7 hips), mixture of autologous slurry and allogenic bone chips (10 hips)	NA	58.7 (28–97.7)	90.6 (61.1–100)

EP, ending point.

**Table 4:** Summary of complications.

Study	No. of hips	Dislocation	PJI	Aseptic loosening		Osteolysis	Superficial infection	Nerve injury	Fracture	DVT	Medical complications
				Acetabular	Femoral						
Zhang 2019	63	1	1	0	0	0	0	NA	1	NA	NA
Zhou 2018	77	4	2	1	NA	0	0	0	0	0	0
Salem 2018	17	0	0	0	0	0	1	0	0	0	NA
Moon 2018	80	3	0	7	NA	7	1	1	NA	NA	3
McLaughlin 2018	61	3	2	1	NA	0	0	NA	NA	1	NA
Jo 2016	51	0	0	4	0	3	1	NA	1	NA	NA
von Roth 2015 and Whaley 2001	89	11	1	5	5	1	0	5	1	NA	1
Gustke 2014	216	9	1	3	NA	NA	NA	NA	NA	NA	NA
Lachiewicz 2013	108	12	4	4	5	2	0	0	3	NA	NA
Wedemeyer 2008	17	1	1	1	NA	0	2	NA	NA	NA	NA
Fan 2008	47	5	1	0	0	0	1	NA	NA	NA	NA
Hendricks 2006 and Dearborn 2000	24	5	5	0	1	1	0	2	3	NA	1
Patel 2003	43	2	0	2	1	0	0	0	0	0	NA
Obenaus 2003	60	0	2	1	0	0	0	NA	NA	NA	NA
Total	953	5.9% 56/953	2.1% 20/953	3.0% 29/953	2.4% 12/502	1.9% 14/720	0.8% 6/720	1.8% 8/438	1.9% 9/472	0.5% 1/196	1.8% 5/270

PJI, periprosthetic joint infection; DVT, deep vein thrombosis.

**Table 5:** Terms of reoperation and revision.

Study	No. of hips	Overall reoperation	Reasons for reoperation						Cup re-revision
			Dislocation	Aseptic loosening	PJI	Osteolysis	Fracture	Others	
Zhang 2019	63	2	0	0	1	0	1	0	1 (1 PJI)
Zhou 2018	77	2	0	1	1 (1.3%)	0	0	0	2 (1 PJI, 1 aseptic loosening)
Salem 2018	17	0	0	0	0	0	0	0	0
Moon 2018	80	2	0	1	0	2	0	0	1 (1 aseptic loosening due to osteolysis)
McLaughlin 2018	61	5	2	1	2	0	0	0	4 (2 PJI, 1 aseptic loosening, 1dislocation)
Jo 2016	51	4	0	4	0	0	0	0	4 (4 aseptic loosening)
von Roth 2015 and Whaley 2001	89	18	4	10 (5 A, 5 F)	1	1	1	1	7 (5 aseptic loosening, 1 PJI, 1 dislocation)
Gustke 2014 and Gustke 2004	216	8	7	3	1	0	0	0	7 (3 aseptic loosening, 3 dislocation, 1 PJI)
Lachiewicz 2013	108	20	3	8 (3 A, 5 F)	4	2 (F)	3	0	7 (3 aseptic loosening, 4 PJI)
Wedemeyer 2008	17	2	0	1	1	0	0	0	2 (1 aseptic loosening, 1 PJI)
Fan 2008	47	3	2	0	1	0	0	0	3 (2 dislocation, 1 PJI)
Hendricks 2006 and Dearborn 2000	24	8	1	0	5	1 (F)	0	1 (nonunion)	5 (5 PJI)
Patel 2003	43	5	1	3 (2 A, 1 F)	0	0	0	1	3 (2 aseptic loosening, 1 dislocation)
Obenaus 2003	60	3	0	1	2	0	0	0	2 (1 aseptic loosening, 1 PJI)
Total	953	82/953 8.6%	20/953 2.1%	34/953 3.6%	19/953 2.0%	6/953 0.6%	5/953 0.5%	3/953 0.3%	48/953 (18 PJIs, 22 aseptic loosenings, 8 dislocations) 5.0%

PJI, periprosthetic joint infection.

**Table 6:** Radiological outcome.

Study	Hips	Vertical position of hip center (mm)			Horizontal position of hip center (mm)			Leg-length discrepancy (mm)	
		Preoperative	Postoperative	Contralateral	Preoperative	Postoperative	Contralateral	Preoperative	Postoperative
Peng 2021	88	NA	23.0 ± 6.1	15.4 ± 3.4	NA	36.4 ± 4.6	35.9 ± 4.4	NA	
Zhang 2019	63	29.7 ± 10.4	22.3 ± 7.6	14.0 ± 3.7	30.8 ± 6.6	29.5 ± 3.7	30.3 ± 3.3	−16.8 ± 17.1	−5.6 ± 11.8
Zhou 2018	77	30.3 ± 10.4	24.1 ± 8.4, +9.6 ± 8.7 relative to the contralateral		30.5 ± 6.9	31.1 ± 4.8, −0.5 ± 8.2 relative to the contralateral		−18.8 ± 16.1	−6.2 ± 15.5
Salem 2018	17	NA			NA			−26 (−20 to 30)	−1 (−5 to 0)
Jo 2016	51	Superior migration of 12.1 ± 4.5 mm in rim fixation group, 11.3 ± 5.6 mm in no rim fixation group			Lateral migration of 3.0 ± 1.3 mm in rim fixation group, 2.3 ± 1.5 mm in no rim fixation group			NA	
Nwankwo 2014	98	NA	32.6 ± 9.6	21.7 ± 8	NA			NA	
Wedemeyer 2008	17	35 ± 8	31 ± 9	NA	34.5 ± 7.2	36.2 ± 6.8	NA	NA	
Fan 2008	47	31	27	NA	NA			NA	
Obenaus 2003	60	NA	11.4 mm (0–41) above anatomic rotation center	NA	NA			NA	
Whaley 2001	89	40 (17–67)	33 (10–58)	NA	39	43	NA	NA	
Dearborn 2000	24	NA	Elevated averagely 4 mm	NA	NA			NA	

### Definition of the jumbo cup

There were two main definitions of the jumbo cup among the studies. One was a diameter >66 mm for males and >62 mm for females and was usually adopted in European and American studies (35, 36, 39–42, 45, 46, 48). The other was a diameter >64 mm for males and >60 mm for females and was usually adopted in Asian studies (31–33, 44). Other definitions included diameter >60 mm (34, 47), >64 mm (43), >65 mm (38), >66 mm (49), and diameter >10 mm than templated contralateral hip (37).

### Clinical analysis

#### Bone defect evaluation

All 14 studies reported the severity of acetabular bone defect. The Paprosky classification was adopted in 13 studies of 881 hips (32–37, 39, 41–44, 46, 47). Paprosky type IIB accounted for the largest proportion (26.6%, 234/881), followed by type IIA (22.5%, 198/881), type IIIA (21.6%, 190/881), type IIC (18.5%, 163/881), type IIIB (7.4%, 65/881), and type I (3.5%, 31/881). The AAOS classification was utilized in five studies with 372 hips (41, 43, 46, 48, 49). AAOS type III occupied the most (62.1%, 231/372), followed by type II (30.1%, 112/372), type I (7.3%, 27/372), and type IV (0.5%, 2/372). In most studies, additional procedures such as structural bone grafting and press-fit bone grafting were also performed to fill the severe acetabular bone defect.

#### Reoperation

All 14 clinical studies reported the rate of reoperation. The overall reoperation rate was 8.6% (82/953). The most common reasons for reoperation were aseptic loosening and dislocation with an incidence of 3.6% and 2.1%, respectively. Removal of the jumbo cup was defined as failure. The failure rate of the jumbo cups was 5.0% (48/953). Among the 48 failed jumbo cups, 22 were removed for aseptic loosening, 18 were removed for periprosthetic joint infection (PJI), and 8 were removed for dislocation. The survivorship of the jumbo cups was 95.0% in the mean follow-up of 9.3 years.

#### Complications

All 14 clinical studies reported complications. Due to the lack of a clear definition of complication, the overall complication rate was not calculated. Dislocation was the most common complication with a rate of 5.9%, followed by aseptic loosening (3.0% in acetabular and 2.4% in femoral) and PJI (2.1%).

#### Dislocation

Dislocation was the most frequent complication with a prevalence of 5.9% (56/953). Of the 56 dislocations, 20 (35.7%) accepted reoperation, with 7 re-revisions of the acetabular cup [five studies (36, 39, 41, 44, 46)], 7 femoral and liner re-revisions [three studies (39, 41, 42)], 4 femoral head and/or liners exchange [four studies (36, 39, 41, 42)], one femoral re-revision [one study (39)], and 1 resection arthroplasty [one study (41)].

#### Aseptic loosening

The incidence of jumbo cup aseptic loosening was 3.0% [29 of 953 hips, 10 studies (33, 35–37, 39, 41–43, 46, 47)]. Among these cases, 22 were managed with acetabular re-revision, 5 refused reoperation, and 2 could not receive re-revision for medical problems.

#### Periprosthetic joint infection

PJI was the third most common complication after dislocation and aseptic loosening, with a rate of 2.1% [20 of 953 hips, 10 studies (32, 33, 36, 38, 39, 41–44, 47)]. Only one patient was treated with antibiotics only for his poor physical condition as he could not tolerate re-revision. The remaining 19 cases of PJI were treated with re-revision and the jumbo cups were removed.

### Harris Hip Score

Eleven studies (687 hips) reported the pre- and postoperative Harris Hip Score (HHS) (32–36, 38, 39, 41, 43, 46, 47). The mean preoperative HHS was 49.4 (poor) and improved to 78.2 (fair) at the latest follow-up.

### Radiological measurements

#### Vertical position of the hip center

Seven studies (461 hips) reported the postoperative vertical distance of hip center elevation relative to the contralateral hip center (31–33, 37, 40, 47, 49). The postrevision hip center was elevated 10.3 mm on average. Five studies (293 hips) compared the pre- and postoperative vertical positions of the hip center (32, 33, 43, 44, 48). Compared with the preoperative condition, the postrevision hip center dropped 6.2 mm on average in the vertical position.

#### Horizontal position of the hip center

Four studies (279 hips) reported the postoperative lateral migration of the hip center relative to the contralateral hip center (31–33, 37). The revision hip center moved 0.4 mm laterally on average. Four studies compared the pre- and postoperative horizontal positions of the hip center (32, 33, 43, 48). The postoperative hip center migrated 1.4 mm laterally on average compared with the preoperative position.

#### Leg-length discrepancy

Three studies reported an improvement of LLD (32–34). The mean LLD was corrected from a preoperative 18.8 mm to a postoperative 5.4 mm.

## Discussion

The jumbo cup has been used for acetabular bone defect reconstruction in rTHA for a long time. To the best of our knowledge, this is the first systematic review to evaluate the evidence of a jumbo cup in rTHA. In general, the results indicated that the jumbo cup was a favorable option for acetabular reconstruction in rTHA for satisfying survivorship and acceptable complication rates.

As was summarized previously, there was no unified definition for the jumbo cup. The definition differed between the studies and could be influenced by the factors of time, race, and surgeon preference. Therefore, to a greater extent, the jumbo cup represents a special idea for acetabular reconstruction. By using a “very large” cup in this study, the contact area with the host bone increased; thereby, the goal of three-point stability and bone ingrowth was achieved.

With respect to postoperative complications, dislocation occupied the first position, with the highest rate of 5.9%. Therefore, we should lay emphasis on this complication and try to place the large diameter femoral head component to reduce the rate of dislocation. We should also identify the patients with high risk of dislocation in advance according to the reported risk factors related to dislocation after rTHA, such as advanced age, history of instability, and prior revision history (50). However, this rate of dislocation (5.9%) is also acceptable because the rate of dislocation is naturally high after rTHA, with 5%–35% in various studies (51–54). In a meta-analysis by Guo et al. that included 4,656 rTHAs, the accumulated incidence of postoperative dislocation was 9.04% (55). Many factors contribute to the high rate of dislocation after rTHA. Due to the extensive exposure in revision arthroplasty, the soft tissue deconstruction process is more severe, especially in revision for PJI because of the thorough debridement procedure. In addition, the posterolateral approach is commonly applied in rTHA, which also leads to injuries of the abduction muscles and an increased risk of dislocation (56, 57). Moreover, the rotation center may not restore in its original position and the offset may also be unsatisfying after rTHA. All these factors may lead to soft tissue imbalance and further postoperative dislocation. However, most dislocations can be addressed by conservative therapy; dislocation is just the second cause for reoperation. Therefore, it is also unnecessary to harbor too much fear for dislocation.

The rate of reoperation was 8.6% and the re-revision of the jumbo cups was 5.0%. Aseptic loosening was the primary mode of failure leading to reoperation and re-revision of the jumbo cups. This may be related to the fixation mechanism of the jumbo cup, which increases the contact area with the host bone to achieve bone ingrowth. Nevertheless, the local host bone may become severely ossified and the bioactivity of the implanted bone is also unreliable. In addition, there is some bone loss in the filing process. These elements can impede bone ingrowth and further lead to a failure of fixation. Hence, if the intraoperative findings indicate that the condition of the local host bone is poor for bone ingrowth, the rotation center elevation technique should be adopted to provide more contact area as well as better bioactivity of the host bone, which is beneficial for bone ingrowth and biomechanical instability.

The radiological outcome indicated that the rotation center position was elevated postoperatively compared with the original anatomical position. This is quite understandable for the combination of the rotation center elevation technique to achieve successful bone ingrowth as mentioned before. Another finding is that the elevation of the rotation center position has already existed preoperatively due to the acetabular bone defect, which also attained a certain degree of correction through the rTHA. The mean postoperative LLD was only 5.4 mm, which is quite acceptable and has no adverse influence on the patient's feeling and limb function. The Harris Hip Score also improved significantly, which indicated that the general condition (pain, activity, deformity, and range of motion) of the hip improved after rTHA. The revised hip could be regarded as meeting the need for daily life, as the mean postoperative HHS was up to the fair level. Thus, although *in situ* reconstruction of the rotation center is the gold standard in rTHA, good clinical results can also be received in the condition of rotation center elevation.

PJI is a disastrous complication that keeps troubling arthroplasty surgeons. For rTHA, the risk of PJI is higher than in primary THA. The rate of PJI in postrevision THA was in the range of 1.3%–17.3% from different registers (58–60). Fröschen et al. retrospectively analyzed 68 rTHAs using custom-made acetabular implants for Paprosky IIIA or IIIB acetabular bone defect reconstruction; the rate of postoperative PJI was unexpectedly up to 22% (61). In systematic reviews of other reconstruction methods for acetabular bone defect, the PJI rates of the custom triflange acetabular component and cup-cage technique were 6.2% and 3.3%, respectively (21, 22). In our systematic review of the jumbo cup for rTHA, the rate of PJI was only 2.1%, which can be regarded as relatively low. We speculate that this reduced rate of PJI may be due to the simplified procedure in acetabular reconstruction of jumbo cups, which may save surgical time and lower the risk of PJI. Another reason is that the jumbo cup method requires a smaller amount of implant compared with other methods, such as porous metallic augments, cup-cage, and monoflanged acetabular component. However, many factors can affect the rate of PJI, such as femoral revision or no revision, use of antibiotics, type of material, and implant coating design. Therefore, we still recommend that this relatively low rate of PJI should be treated with caution because of some existing bias and the relatively simple statistical method.

There are also some controversies on using the jumbo cup for rTHA. In 2016, Lachiewicz and Watters pointed out that although the jumbo cup had shown excellent 10-year survivorship, the late loosening of “first-generation” porous surfaces and wear with periprosthetic osteolysis of traditional polyethylene liners had also been reported and needed more attention (27). They also recommended the use of enhanced porous coatings, highly cross-linked polyethylene liners, and large femoral heads in jumbo cup reconstruction. Zhou et al. retrospectively compared 74 consecutive rTHAs using metal augments with a cementless hemispherical cup and 77 consecutive rTHAs using the jumbo cup (33). The biomechanical parameters of the metal augment group, such as rotator center position, leg-length discrepancy, head-cup difference, and femoral offset, are all superior to those of the jumbo cup group. In recent years, many advanced reconstruction techniques such as 3D printing, custom prosthesis, and robot-assisted arthroplasty have been developed and seem to stand for the future direction of rTHA (62–64). Even so, the results of our systematic review have already supported the jumbo cup as a successful method for acetabular reconstruction in rTHA. The development of advanced biomaterials will further improve the performance of the jumbo cup.

Our study also has several limitations, some of which are listed here. First, the quality of the included studies is relatively low, and most of these are of a single-arm design. Second, the sample size is also relatively limited. In addition, the acetabular bone defects of many included cases are mild-to-moderate and relatively easy to reconstruct. In fact, the reconstruction for severe acetabular bone defect is the real hot spot and core problem in rTHA. Therefore, if only the performance of the jumbo cup for severe acetabular defect is discussed, the results may not be presented as well as those in our study. Unfortunately, most papers report the overall survivorship and failure and do not report the relation with the extent of bone defect. Therefore, this relation was not explored in this systematic review. Certainly, on the other hand, the results can also indicate that the jumbo cup is a good option for mild-to-moderate acetabular bone defect at least.

## Conclusion

In summary, according to this systematic review, the jumbo cup is a recommended method for acetabular reconstruction in rTHA. The clinical outcomes and survivorship of the jumbo cup are satisfying. However, in most cases, the acetabular bone defects are mild to moderate. Further research is still required to review its performance for severe or extreme acetabular bone defect reconstruction.

## Data Availability

The original contributions presented in the study are included in the article/Supplementary Material, further inquiries can be directed to the corresponding authors.
